# Topics of the Month

**Published:** 1892-07

**Authors:** 


### TOPICS OF THE MONTH.

THE JENNIE CASSEDAY FREE INFIRMARY FOR WOMEN, of Louisville, Ky., was formally opened for the reception of patients, April 12, 1892. The object of this infirmary is the treatment of the diseases peculiar to women, and it was founded, is owned and conducted by the order of King's Daughters, in Louisville, Ky. Dr. Lewis S. McMurtry is the attending surgeon, and the patients are placed under his skilful surgical supervision, which has already made an astonishing record of success. At the present writing, there are several patients in the private rooms as well as in the free ward, that have undergone abdominal section, and are already passing towards convalescence and assured recovery. This is, so far as we know, the first institution of this kind organized by the order of King's Daughters, and this appears to us one of the most satisfactory methods of making this noted organization useful to society.

The Board of lady managers of this infirmary is presided over by Miss Jennie C. Benedict, Chairman, and it would be difficult to find a more competent young woman for this responsible and arduous place. A recent visit to the infirmary and a careful inspection of the buildings and grounds warrant us in saying that it is one of the most completely appointed institutions of its kind in this country.

---

THE FOOD MANUFACTURERS' ASSOCIATION will, as we have heretofore announced, hold an exposition at Madison Square Garden, New York, in October next. The announcement is made that Miss Maria Parloa, the celebrated teacher and authority in the art of cooking, will take charge of that department in the exposition referred to. Miss Parloa will lecture and give practical demonstration in cooking every afternoon during the exposition in the Concert Hall. This, together with the Seidl orchestra, led by Herr Anthon Seidl in person, will prove an attractive feature of the exposition.

---

FOLLOWING close upon the announced intention of a number of our philanthropic citizens to establish a cholera infantum hospital in the vicinage of Buffalo, comes now the announcement that a children's hospital, for the care and treatment of sick, injured, and crippled children, has already incorporated. The names of the incorporators and the Board of Managers we cheerfully give below: The Board of Managers for the first year are Sara L. Truscott, Florence D. Folwell, Ottilie H. Rochester, Elizabeth S. Parkhurst, Martha T. Williams, Margaret L. P. Adam, Kate L. Hamlin, Katherine B. Lewis, and Annie W. Spaulding. The other incorporators are Henry M. Watson, Henry W. Sprague, Bernard Bartow, Frances R. Hunsicker, Sherman S. Rogers, Fannie F. Bartow, Geneva L. Wheeler, Ellen O. Watson, George L. Williams, Susan F. Rumsey, M. B. Folwell, and Harriet C. Williams.

There is no more useful charity than one that has for its purpose the cure of crippled children. We have a tender corner in our heart for anyone who is instrumental in rescuing one of these maimed little sufferers from the jaws of crippled invalidism, and in bringing them into the realms of self-supporting happiness.

---

The administration of the Health Department of the Port of New York, under Ex-Health Officer William M. Smith, who held the office for twelve years, must have been very satisfactory indeed to all who came in official contact with the health officer, judging from the numerous expressions of gratitude that have come from several sources since the office changed hands. The Chamber of Commerce of the State of New York and the Maritime Association of the Port of New York have passed memorials commending the administration of Dr. Smith, while the shipping men, through the agents of the several steamship lines running to the port, have presented him with a handsome testimonial, in the nature of engrossed resolutions in an album especially designed for the purpose, besides twenty-five water-color sketches, representing ships in mid-ocean, and illustrating the health officer's duties in examining incoming vessels. The resolutions were signed by all the leading owners and agents of the principal steamship lines running into the port of New York.

---

The Medical Society of the County of Erie signalized the severance of its affiliation with the American Medical Association, which was done by vote of the Judicial Council at Detroit, in holding one of the best meetings, considered from a standpoint of scientific work, that has fallen to its lot in many years. This meeting, held June 14, 1892, was not only one well attended, but its sessions were prolonged beyond the usual hours. The profession of Buffalo and vicinity were highly favored in having in attendance four or five distinguished men from distant cities, who read papers and participated in the debates. The JOURNAL hopes to present to its readers a full report of this meeting by printing the papers and the discussions in an early number. The guests were one and all united in their praise of Buffalo and the royal treatment they received at the hands of the honorable profession of medicine of the Queen City of the Lakes.

---

WE TAKE pleasure in directing attention to the advertisement of the Buffalo Medical College. This school will endeavor to furnish its students, the coming session, with clinical instruction in obstetrics. This movement is in the right direction, and shows an appreciation of the important work in the Niagara Medical College, the obstetric clinic of which is inferior to none other in this country. We wish success to the effort, inasmuch as it is a movement too long delayed, but of such vast importance to the student in medicine, that it should receive the encouragement of every friend of the college.

---

THE election of officers for the Buffalo Academy of Medicine was held Tuesday afternoon, June 21st, at the Y. M. C. A. Lecture-room, and resulted as follows: President, DeLancey Rochester; Secretary, William C. Krauss; Treasurer, Eugene A. Smith; Trustees, J. W. Putnam, A. Dagenais, R. Park. A meeting of the Academy was held in the evening, and the above-mentioned officers duly installed. The interest manifested in the Academy by the professions shows that the project is meeting with all the enthusiasm expected of it.

---

THE NEW YORK PHYSICIANS' MUTUAL AID ASSOCIATION, in which so many of our readers cannot fail to possess an interest, has reached a point in membership where it will henceforth be able to pay $1,000 upon the death of a member, which is the full limit allowed by the by-laws. A further increase of membership is, however, desirable, because it will serve to reduce the assessments, even though they are now comparatively small, being very much less than that of any other associated membership which has an insurance clause. Doctors are very slow to attend to affairs like these, as compared with other pursuits, but when an association offers the advantages and protection that can be obtained by membership in this our New York organization, we are amazed at some of the excuses offered for not uniting with it. Applications may be made to the local treasurer, Dr. W. G. Gregory, 530 Main street, Buffalo, N. Y.

---

Dr. A. A. Hubbell, of Buffalo, is the fortunate possessor of a complete set of the oldest ophthalmic journal in existence, viz., *Annales d' Oculistique.* It was founded in 1838 by Currier, and continued by Warlomont till his death in 1891. Since then its office of publication has been removed from Brussels to Paris, where it is ably conducted by Valude. It reflects most completely the progress of ophthalmology throughout the world during the past fifty-five years, and has been, and continues to be, a powerful factor in promoting the scientific advances in this department of medicine. The set which Dr. Hubbell has been so fortunate to obtain has the additional interest of having belonged to the late Dr. Warlomont himself, who for half a century was its distinguished editor.

---

The American Medical Association, at its recent meeting in Detroit, adopted a resolution, introduced by Dr. Charles A. L. Reed, of Cincinnati, looking to the annexation of Canada, at least the medical portion thereof, to the United States. In other words, should this amendment to the by-laws prevail, the Dominion of Canada will be embraced in the jurisdiction of the American Medical Association, and we may soon expect to see the Canadian profession walking arm-in-arm with our own down the aisles at the meetings. At the Detroit meeting, many distinguished physicians from Canada attended by invitation, and took part in the work of the sections. It looks as though they would soon take their places as members, with all the rights and privileges pertaining to those from the United States, to which they will assuredly be very welcome.

---

With this July number the Journal closes Volume XXXI. We present herewith a comprehensive index, which we hope our readers will carefully examine. It exhibits a résumé of our work for the year, and we submit it in the hope that it will merit their approval and bring to us further aid and comfort in the conduct of our difficult and painstaking enterprise, in the way of new subscribers and encouraging words. For the future, we can only say that we expect to maintain the JOURNAL on the basis of its present lines, and to improve it from time to time, as fast as our increasing patronage will permit.

---

AT THE National Conference of Delegates of the State Medical Examining and Licensing Boards, held in Detroit, June 8 and 9, 1892, a permanent organization was effected. Dr. William Perry Watson, chairman of the committee, reported a constitution and by-laws, which were considered and adopted with some minor modifications, and the following officers were elected for the ensuing year, namely: President, Dr. John H. Rauch, of Chicago, Ill.; Vice-President, Dr. William Warren Potter, of Buffalo, N. Y.; Secretary and Treasurer, Dr. Hugh M. Taylor, of Richmond, Va. It is intended to hold annual meetings of this organization in connection with the regular meetings of the American Medical Association, with the hope of bringing the different medical examining and licensing boards into closer relation, and to exchange views and opinions that have grown out of the accumulated experience of the past, as well as to promote and encourage the organization of similar boards in states where they do not exist.

